# BALANCE Dietary Index in Patients with Heart Failure, and Its Adherence in Sergipe, Brazil

**DOI:** 10.3390/clinpract12030043

**Published:** 2022-05-31

**Authors:** Jamille Oliveira Costa, Felipe J. Aidar, Juliana Santos Barbosa, Luciana Vieira Sousa Alves, Victor Batista Oliveira, Larissa Marina Santana Mendonça de Oliveira, Raysa Manuelle Santos Rocha, Diva Aliete dos Santos Vieira, Ingrid Maria Novais Barros de Carvalho Costa, Márcia Ferreira Cândido de Souza, Joselina Luzia Menezes Oliveira, Leonardo Baumworcel, Eduardo Borba Neves, Alfonso López Díaz-de-Durana, Marcos Antonio Almeida-Santos, Antônio Carlos Sobral Sousa

**Affiliations:** 1 Graduate Program in Health Sciences, Federal University of Sergipe (UFS), Aracaju 49060-676, SE, Brazil; barbosa.juliana@live.com (J.S.B.); lucianaalvesnutri@gmail.com (L.V.S.A.); vbo.nutri@gmail.com (V.B.O.); nutrilarissamarina@gmail.com (L.M.S.M.d.O.); ysamanu@hotmail.com (R.M.S.R.); joselinamenezes@gmail.com (J.L.M.O.); acssousa@terra.com.br (A.C.S.S.); 2 Group of Studies and Research in Performance, Sport, Health and Paralympic Sports—GEPEPS, Federal University of Sergipe (UFS), São Cristóvão 49100-000, SE, Brazil; fjaidar@academico.ufs.br; 3 Graduate Program in Physical Education, Federal University of Sergipe (UFS), São Cristóvão 49100-000, SE, Brazil; 4 Graduate Program in Physiological Science, Federal University of Sergipe (UFS), São Cristóvão 49100-000, SE, Brazil; 5 Department of Nutrition, Campus Prof. Antônio Garcia Filho, Federal University of Sergipe (UFS), Lagarto 49400-000, SE, Brazil; divaaliete@academico.ufs.br; 6 Federal Institute of Sergipe, São Cristóvão Campus, Food Technology Coordination, São Cristóvão 49100-000, SE, Brazil; ingrid_novais@infonet.com.br; 7 Graduate Program Professional in Management and Technological Innovation in Health, Federal University of Sergipe, Aracaju 49100-000, SE, Brazil; nutrimarciacandido@gmail.com; 8 Department of Medicine, Federal University of Sergipe (UFS), São Cristóvão 49100-000, SE, Brazil; 9 Division of Cardiology, University Hospital of Federal University of Sergipe (UFS), São Cristóvão 49100-000, SE, Brazil; 10 Clinic and Hospital São Lucas/Rede D’Or São Luiz, Aracaju 49060-676, SE, Brazil; leonardo.baumworcel@caxiasdor.com.br (L.B.); marcosalmeida2010@yahoo.com.br (M.A.A.-S.); 11 Graduate Program in Biomedical Engineering, Federal Technological University of Paraná (UTFPR), Curitiba 80230-901, PR, Brazil; eduardoneves@utfpr.edu.br; 12 Sports Department, Physical Activity and Sports Faculty-INEF, Universidad Politécnica de Madrid, 28040 Madrid, Spain; alfonso.lopez@upm.es; 13 Graduate Program in Health and Environment, Tiradentes University (UNIT), Aracaju 49010-390, SE, Brazil

**Keywords:** dietary patterns, cardiovascular diseases, cardiac insufficiency, food consumption, quality of life

## Abstract

Background: “The effective treatment of Heart Failure (HF) involves care with food intake. Recently, the Ministry of Health created the Brazilian Cardioprotective Diet and its dietary index, BALANCE, which assesses adherence to the standard’s recommendations”. Methods: This observational prospective study is part of the Congestive Heart Failure Registry (VICTIM-CHF) of Aracaju/SE. Observations and data collection took place from April 2018 to February 2021. Sociodemographic and clinical aspects and food consumption were evaluated. Food intake was determined using the food frequency questionnaire. Foods were categorized using the BALANCE dietary index into green, yellow, blue and red food groups. The BALANCE dietary index was obtained using median and interquartile ranges, scores of the Mann–Whitney U test, and associations between clinical variables and the index, through linear regression. Results: Participants included 240 patients with HF (61.12 ± 1.06 years), who were assisted by the Unified Health System (67.5%). Individuals with a partner showed greater adherence to the green food group recommendations (0.09; 0.00–0.17). The lowest adherence to recommendations regarding the blue food group was observed in individuals with excess weight, who had a higher consumption of foods rich in animal protein (0.54; 0.38–0.78). As for the red food group (ultra-processed foods) the highest adherence was observed by patients with diabetes mellitus (0.41; 0.05–0.77). The greatest adherence to the yellow food group, and a higher score, was observed in patients with the smallest left ventricular systolic diameter (LVSD). Conclusions: Being married was directly associated with the consumption of foods in the green group, while being overweight and having diabetes were inversely associated with adherence to the blue and red food groups, respectively. Greater adherence to the yellow food group recommendations was inversely associated with less change in the DSFVE.

## 1. Introduction

Cardiovascular diseases (CVD) continue to be leading causes of death; heart failure (HF) has a significant influence on these rates [1]. Because it is present worldwide, with a mortality rate between 10 and 13.4% [2,3,4] and a cost between USD 22.1 and USD 30.7 billion to health systems [3,4,5], the World Health Organization (WHO) asks countries to prioritize combating this clinical syndrome marked by the inability of the heart to pump blood and nutrients to the metabolic tissues [6,7,8]. If nothing is done, it is projected that by 2030 there will be a 46% increase in the incidence rate, which will result in eight million people diagnosed with HF being held hostage by their physical and emotional limitations [9,10,11,12].

One of the measures necessary to improve the quality of life of people affected by this disease is the creation of strategies that encourage the adoption of healthy eating habits that promote improved cardiovascular function and positively impact morbidity and mortality rates [13,14,15]. Some dietary indices, based on nutritional recommendations or guidelines already present in the literature, demonstrate effective results between healthy eating and improved clinical prognosis [4,16,17,18,19,20,21]. However, these dietary patterns often contain foods that are not regional, or do not belong to the local culture, and are difficult to access, which creates barriers and makes adherence difficult.

To address this, the Ministry of Health, in partnership with Heart Hospital (HCor), adapted the recommendations of dietary standards and clinical guidelines with the objective of increasing the consumption of regional foods with a protective function for cardiovascular health; the resulting Brazilian Cardioprotective Diet had its efficacy tested in a cardioprotective nutrition education program: BALANCE. This new standard categorizes foods according to their nutritional composition into four groups represented using the colors of the Brazilian flag (green, yellow and blue), plus red. The proportion of these colors on the flag indicates the frequency with which each food group should be consumed to be considered protective of cardiovascular outcomes. Green is the predominant color on the flag, indicating that foods from the green group should be consumed most frequently, while those from the yellow group should be consumed in moderation, and foods from the blue group should be consumed occasionally. The red group represents ultra-processed foods that are not recommended in this food pattern; there is no red on the Brazilian flag. This standard is recommended for individuals who are overweight or obese and/or have controlled systemic arterial hypertension, compensated type 2 diabetes mellitus, dyslipidemias and/or CVD [22].

With the objective of evaluating the effectiveness of the nutritional recommendations of the Brazilian Cardioprotective Standard and cardiovascular outcomes, Silva et al. [23] created the BALANCE dietary index. However, because it is a new standard and index, there are few studies that associate it with the prevention or treatment of the diseases for which it is recommended. Therefore, the present study aims to evaluate the level of general and per group adherence to the BALANCE dietary index by hospitalized patients with HF, and the relationship between adherence and demographic, economic, and clinical factors, as well as the type of hospital care provided in Sergipe, Brazil.

## 2. Materials and Methods

### 2.1. Study Design

The study is cross-sectional and was carried out from April 2018 to February 2021 in four referral hospitals (2 public and 2 private) in cardiology in Aracaju/SE. It is part of the Congestive Heart Failure Registry (VICTIM-CHF) of that state.

### 2.2. Participants

Individuals of both sexes aged over 18 years who were hospitalized for HF decompensation participated in the study. Diagnosis and admission to the research study were performed using the Boston Score and Framingham criteria by two examiners from the hospital’s cardiology team [24]. Only participants who maintained this diagnosis at hospital discharge remained in the survey. Exclusion criteria included the presence of other chronic debilitating diseases (such as the human immunodeficiency virus, cancer or chronic obstructive pulmonary disease), difficulty in oral feeding and psychiatric or neurocognitive conditions.

As the study is part of a registry, all participating hospitalized patients signed the free and informed consent form. The study was approved by the Research Ethics Committee of the Federal University of Sergipe (CAAE: 87651117.8.0000.5546; Opinion: 2,670,347).

### 2.3. Sociodemographic, Clinical and Lifestyle Data

The identification variables (age, sex, race, schooling, family income, presence of associated pathologies, type and etiological factor of HF and functional capacity quantified by the New York Heart Association (NYHA), which stratifies by the degree of physical limitation into classes I, II, III and IV [25]) were collected using interviews as well as analysis of medical records.

Doppler echocardiography (ECO) performed at the inpatient hospital was used to assess clinical cardiac function. The variables analyzed were left ventricular ejection fraction (LVEF), categorized as preserved (>50%) and altered (<49%); left ventricular end-systolic (LVESD) diameters; and left ventricular end-diastolic (LVEDD) diameters. Clinical outcome at 30 days was classified as with or without uneventful outcomes (death, readmission, urgency, or surgery). 

Participants were asked about alcohol consumption and smoking. Regular practice and intensity of physical activity were evaluated using the International Physical Activity Questionnaire (IPAQ), an instrument recommended by the World Health Organization (1998) and validated in Brazil by the Study Center of the Physical Aptitude Laboratory of São Caetano do Sul—CELAFISCS [26].

### 2.4. Anthropometric Data

After stabilization of the patient in the hospital, weight and height were measured in triplicate by the previously trained nutrition team to minimize errors. Height was measured using a stadiometer (Seca ®, Hamburg, Germany), with markings in millimeters. To measure body weight, an electronic digital scale was used, with a maximum capacity of 180 kilograms and an approximation of 100 grams (Seca ®, Hamburg, Germany).

The body mass index (BMI) was calculated as the ratio between body weight (kg) and height squared (m) and classified based on the cutoff points proposed by the Ministry of Health [27,28].

### 2.5. Food Data

Food intake was assessed by applying the semi-quantitative Food Frequency Questionnaire (FFQ), with 81 foods adapted for the population under study, using the instrument developed by Furlan-Viebig and Pastor-Valero [29] to assess the relationship between diet and disease. The frequency of food intake questions (daily, weekly, monthly, yearly or never) referred to the past year.

To minimize limitations such as the definition of the quantity of food consumed, some methodological precautions were adopted, such as the use of visual resources (including a photo album, utensils and standard measures). In addition, interviewers were trained, a pilot test was carried out to resolve doubts, and homemade measures were standardized.

### 2.6. BALANCE Dietary Index

The assessment of the quality of daily food intake was performed using the BALANCE dietary index, a priori method created by Silva et al. [23].

**Figure 1:**
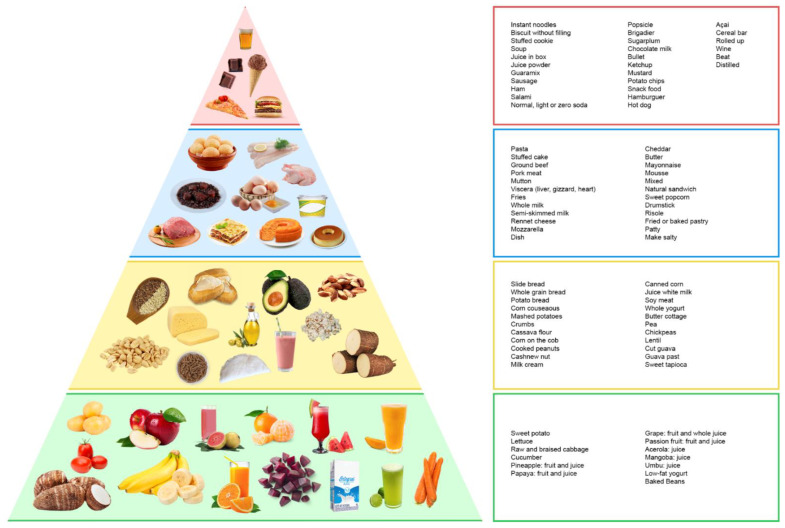
Food groups according to the BALANCE dietary index classification.

To determine the score, foods were initially divided into groups as shown in Figure 1. The food pyramid arranges foods in descending order of consumption from the bottom. Foods that should be consumed in greatest proportion, the green foods, are concentrated in the base, followed by the yellow foods, blue foods, and at the top, red foods, which should be consumed much less frequently. The number of servings consumed was calculated by dividing the amount ingested by values established in food guides and nutritional composition tables. As BALANCE uses the value of energy intake to define the number of servings recommended in each food group, the estimated energy requirement (EER) was calculated using 20 Kcal/Kg of weight for overweight individuals, 25 Kcal for eutrophic individuals and 32 Kcal for individuals with low weights. Individuals who presented EER below the minimum limit of 1400 Kcal or above the maximum limit of 2400 Kcal were evaluated according to the recommendation for their respective groups. Finally, scores were assigned according to overall intake as well as by food group. The overall index score ranged from 0 to 40 points: 0 points were assigned to those who consumed a number of servings different from those recommended; 10 points were assigned to those who consumed the amount indicated by the diet according to the EER.

Zero points were assigned to individuals who did not consume the recommended amount from the green food group (cardioprotective foods); who consumed 50% above or below the recommended amount from the yellow food group (foods that deserve attention in the management of CVD); who ate at least 2 servings more than recommended from the blue food group (harmful foods) and 4 or more servings from the red food group (ultra-processed foods). A score of 10 was assigned to patients who consumed an amount equal to or higher than recommended from the green food group, exactly as recommended for the yellow food group and equal to or lower than recommended for the blue and red food groups. As the blue and red food groups restrict daily consumption, they received an inverse score (the higher the intake, the lower the score). Proportional scoring was performed for participants who ingested intermediate portions.

### 2.7. Statistical Analysis

Generalized linear models (GLM) with link log and Gamma distribution were used to examine the relationship between the total BALANCE score, the green food group score, the yellow food group score and the red food group score, and demographic, socioeconomic and lifestyle characteristics, clinical factors and the type of hospital care provided. The relationship between the blue food group score and independent variables was determined using zero-inflated Poisson regression. Logistic regression was performed to assess the association between the clinical variables and the index, considering the NYHA, ejection fraction and outcome at 30 days as the dependent variables; linear regression was performed with LVSD and LVDD as dependent variables. Data analysis was performed using R software version 3.2.2 (Auckland, New Zealand), public domain; a *p* value < 0.05 was considered significant.

## 3. Results

A total of 240 patients hospitalized for HF decompensation participated in the study, most of them elderly (mean age 61.12 ± 1.06 years) and male (52.08%). The participants were also predominantly self-declared black (73.28%), living with their partners (53.62%), living on a family income of less than one minimum wage (68.33%) and admitted to hospitals of the Unified Health System, Health-SUS (67.50%). Regarding clinical characteristics, patients had altered functional capacity (87.50%), were hypertensive (70.17%), overweight (48.47%) and practiced light to moderate physical activity (84.62%). Alcohol consumption (14.70%) and smoking (4.62%) were not prevalent in the participant population. 

Adherence to a cardioprotective diet was considered low in the participant population, with scores of 14.83 (±0.37), 8.14 (±0.16), 2.60 (±0.22), 1.30 (±0.20) and 2.78 (±0.19) for the diet in general and for the green, yellow, blue and red food groups, respectively. Table 1 presents the socioeconomic, clinical and lifestyle factors associated with the total and food group scores of the BALANCE dietary index. There was a positive association between individuals living with a partner and increased adherence to the green food group recommendations. The lowest adherence to recommendations regarding the blue food group was observed in overweight individuals, who had a higher consumption of foods rich in animal protein. It was observed that participants with diabetes mellitus adhered most closely to the BALANCE recommendations for the red food group, with a lower consumption of processed and ultra-processed foods. The association between the general BALANCE dietary index and groups according to clinical variables is described in Table 2. It was observed that a greater adherence to recommendations for the yellow food group (a higher score) is associated with a lower LVSD.

## 4. Discussion

Effective HF treatment involves careful dietary intake. This requires understanding how food and nutrition influence the clinical prognosis of patients. The literature indicates that this relationship should be studied using dietary patterns which represent the synergistic and antagonistic effects of nutrients and prescribe the type of foods and beverages ingested in different preparations and combinations [30,31].

In general, the population of the present study had low adherence scores to the Brazilian Cardioprotective Diet, with a high median score of 15.53 out of a total possible score of 40. The high adherence score for the green food group may be associated with the fact that the methodology did not assign a penalty for excessive consumption, as the green food group is composed of foods that are sources of vitamins, minerals, fiber and antioxidants considered cardioprotective. The BALANCE educational program intentionally encourages consumption from the green food group, which is currently low in the population of the present study [32,33]. The low scores and high consumption rates for the yellow, blue and red food groups is significant; these groups include foods that can be harmful to cardiovascular health.

The BALANCE dietary index is new and there are few studies to date that evaluate its relationship with the prevention or treatment of cardiovascular diseases; however, it is a combination and adaptation of the Mediterranean and DASH dietary patterns (which have an effective role in cardiovascular disease treatment and prevention, as elucidated in the literature) to Brazilian food culture, so it is important that participants adhere to it [16,17,18,19,20,21]. Weber et al. [34] evaluated the efficacy of the BALANCE dietary index on events and death in patients with established CVD. However, they found that there was no significant effect on the assessed outcome. The authors cited study limitations including the low adherence to the standard and the time and loss of follow-up, which occurred with the most critically ill patients.

In the present study, increased adherence to the green food group recommendations by participants who live with a partner corroborates findings in the literature, which also points to consumption of a better quality of food by people who live with a partner [35,36,37,38]. They had a higher intake of natural or minimally processed foods such as fruits, vegetables and milk, which are present in the BALANCE pattern’s green food group, are considered cardioprotective, and should be ingested more frequently. Alkerwi et al. [39], when evaluating Luxembourgish women, observed a lower quality of diet for single, divorced and widowed women and suggested that this group’s lack of family support and limited financial resources may be associated with restricted access to a variety of food choices. Whitelock and Ensaff [40], when exploring the perceptions and practices of the elderly related to eating behavior and food choices, reported participants’ statements that cooking for oneself generates boredom and reduces motivation when compared to cooking for the family, which increases consumption of ready meals. In addition, eating alone makes the meal less pleasant compared to eating with other people.

The consumption of plant foods (the green food group) has a positive relationship with a favorable prognosis for patients with CVD. This relationship is due to the presence of phytochemicals in foods with potent antioxidant and anti-inflammatory effects [41,42,43]. The most consumed foods in the green food group were bananas, beans, tomatoes, mangoes, oranges, passion fruit, apples, potatoes, pineapples and watermelon. Razavi et al. [44] observed that the intake of diets rich in foods of animal origin and low in vegetables is associated with an increased risk for left ventricular diastolic dysfunction, an alteration associated with mortality.

The high energy density from proteins of animal origin, which are part of the blue food group, may explain the lower adherence to the BALANCE recommendations and the higher consumption of foods such as meat and eggs, milk and dairy products by overweight individuals. Despite the scientific literature illustrating the beneficial effects of protein intake, when separated according to food sources, a high intake of foods of animal origin is associated with an increase in obesity and its comorbidities [39,45]. The physiological relationship is still unclear. The preparation method and the amount of protein consumed are influential factors; the highest daily protein intake tends to be associated with obesity, which is not observed with a low intake [45]. The restriction suggested by the cardioprotective diet for foods in the blue group occurs because animal proteins are sources of saturated fat; an excess of saturated fat is detrimental heart health [22,46]. The Dietary Acute and Chronic Heart Failure Guideline recommends diets that avoid excessive intake of saturated fats and trans fats and that prioritize mono and polyunsaturated fat sources [15]. The blue group foods most often consumed in the present study were coalho cheese, whole milk powder, boiled chicken, whole UHT milk, margarine, fried egg, butter, crumbs, boiled egg and plain cake. 

Concern and health care may be the main reasons patients with diabetes presented direct adherence (lower consumption) to recommendations regarding the red food group. These patients are more likely to be followed up on a regular basis by health professionals, and to receive guidance on care—which leads to greater adherence to treatment. Foods in the red group should be excluded from cardioprotective nutrition, so much so that the group is represented by a color that does not exist on the Brazilian flag. It includes ultra-processed foods that contain high amounts of refined sugars, saturated and trans fats, stabilizers, dyes, sweeteners, flavorings and sodium—all considered harmful to the heart [22]. The main nutritional guidelines and dietary patterns used in the prevention or treatment of diabetes, hypertension and cardiovascular health exclude or restrict the consumption of these foods [47,48,49,50]. In addition to being considered nutritionally unbalanced, they also contain toxic substances produced during processing that directly affect cardiovascular health, such as acrylamide, which is formed at high temperatures [51]; acrolein, a compound formed when fat is heated [52]; bisphenol, which is present in food packaging [53]; and advanced glycation end products (AGE) that can accelerate the development of vascular diseases [54]. 

The Guideline on Fat Consumption and Cardiovascular Health and the Positioning on Fat Consumption and Cardiovascular Health discuss the contribution of fats and simple carbohydrates to the genesis of CVD. According to these documents, high consumption of saturated and trans lipids is correlated with an increase in LDL-c and, consequently, with an increase in cardiovascular risk. In addition, the type of fat consumed can also influence other risk factors such as insulin resistance and increased blood pressure. As for fast-absorbing simple carbohydrates, high intake favors an imbalance between the supply of lipids and other nutrients, allowing the establishment of hypercholesterolemia, excess weight, and the development of obesity and postprandial changes such as hyperglycemia, hyperinsulinemia and hypertriglyceridemia, which are all associated with increased cardiovascular risk [51,55]. In the present study, red group foods with the highest consumption rate were candy, soup, soda, beer, cream crackers, water and salt, stuffed cookies, ice cream, ham and powdered juice.

Studies show that high consumption of ultra-processed foods increases CVD mortality rates [56,57]. Blanco-Rojo et al. [58] found in a Spanish cohort, where high consumption of ultra-processed foods was associated with higher mortality, that the theoretical isocaloric replacement of ultra-processed foods with unprocessed foods will significantly reduce mortality, but not linearly. Moreira et al. [59] estimated that if there is a 50% reduction in ultra-processed foods and a 50% increase in culinary ingredients, Brazil’s mortality rate will experience a decrease of 11% by 2030.

The relationship between the LVSD and the consumption of foods in the yellow group deserves attention, since changes in this marker were associated with cardiovascular mortality [60]. Foods that are part of the yellow group are sources of calories, carbohydrates, minerals, vitamins and sodium that can be harmful in the case of excessive consumption. The yellow group foods most consumed by study participants were white rice, French bread, cassava flour, olive oil, couscous, butter curd, cassava, vitamins, brown rice and pasta. In the present study, it was observed that the greater the adherence to the yellow group’s recommendations, the lower the LVESD. The high intake of fast-absorbing simple carbohydrates favors an imbalance between the supply of lipids and other nutrients, allowing the establishment of hypercholesterolemia, excess weight, the development of obesity and postprandial changes such as hyperglycemia, hyperinsulinemia and hypertriglyceridemia, all associated with insulin resistance and increased cardiovascular risk. Furthermore, this surplus can synthesize saturated fat through de novo lipogenesis [50,60,61]. 

Finally, a sedentary lifestyle or the practice of low or moderate intensity physical activity, as found with higher prevalence in the present study, can compromise the quality of participants’ lives. According to Roscani et al. [62], the practice of resistance exercise associated with aerobic activities promotes improvements in functional classification and, consequently, in a reduction in mortality/hospitalization. Given this, it is clear that health education is important to encourage self-care not only regarding food, but also regarding physical activity to improve quality of life. In a systematic review and meta-analysis, Son, Choi and Lee [63], when evaluating the effectiveness of self-care education performed by nurses for patients with HF, observed a significant reduction in the risk of readmission for all causes (RR = 0.75, 95% CI = 0.66–0.85), HF-specific readmission (RR = 0.60, 95% CI = 0.42–0.85), and all-cause mortality or readmission (RR = 0.71, 95% CI = 0.61–0.82).

The limitations of the present study that must be considered are common to cross-sectional studies that assess food consumption in the population. They are: loss of data for some variables or incomplete information; and bias of over or underestimation using instruments available in the literature that require an interviewee to have an accurate memory. 

Authors should discuss the results and how they can be interpreted from the perspective of previous studies and working hypotheses. The findings and their implications should be discussed in the broadest context possible. Future research directions may also be recommended.

## 5. Conclusions

Low adherence to the BALANCE dietary index was verified in general, and for each of its component food groups, with socioeconomic, clinical and nutritional status factors. There was a direct positive association between individuals living with a partner and a high adherence score for the green food group, while being overweight and having diabetes were inversely associated with adherence to recommendations for the blue and red food groups, respectively. In addition, greater adherence to the yellow food group recommendations was inversely associated with less change in the DSFVE.

## Figures and Tables

**Table 1:** Total and group scores for the BALANCE dietary index according to sociodemographic, clinical, lifestyle and nutritional characteristics of patients hospitalized with CHF in Aracaju, SE, in 2021.

Variables	Adherence		Green Group		Yellow Group		Blue Group		Red Group	
	B *	IC95	B *	IC95	B *	IC95	IRR **	IC95	B *	IC95
**Age in years (*n* = 239)**	0.00	−0.00; 0.01	0.00	−0.00; 0.00	−0.00	−0.03; 0.02	1.00	0.99; 1.01	0.00	−0.00; 0.02
**Gender (*n* = 239)**										
Male (*n* = 124)	Ref.		Ref.		Ref.		Ref.		Ref.	
Female (*n* = 115)	0.09	−0.02; 0.22	0.06	−0.02; 0.15	−0.00	−0.83; 0.81	1.27	0.87; 1.01	−0.02	−0.36; 0.31
**Race (*n* = 231)**										
Not black (*n* = 61)	Ref.		Ref.		Ref.		Ref.		Ref.	
Black (*n* = 170)	−0.03	−0.16; 0.10	0.01	−0.08; 0.11	−0.15	−1.13; 0.82	1.05	0.72; 1.52	−0.06	−0.45; 0.32
**Marital Status (*n* = 234)**										
Single (*n* = 108)	Ref.		Ref.		Ref.		Ref.		Ref.	
With partner (*n* = 126)	0.00	−0.11; 0.12	0.09	0.00; 0.17	−0.58	−1.42; 0.25	0.91	0.65; 1.25	0.09	−0.24; 0.43
**Income (*n* = 239)**										
<1 MW (*n* = 164)	Ref.		Ref.		Ref.		Ref.		Ref.	
≥1 MW (*n* = 75)	−0.06	−0.21; 0,09	−0.07	−0.19; 0.04	−0.09	−1.20; 1.02	0.98	0.67; 1.42	−0.06	−0.45; 0.32
**Type of service (*n* = 239)**										
Private (*n* = 77)	Ref.		Ref.		Ref.		Ref.		Ref.	
Public (*n* = 162)	−0.05	−0.22; 0.10	0.01	−0.10; 0.14	−0.60	−1.70; 0.49	0.90	0.56; 1.45	−0.35	−0.87; 0.15
**Hypertension (*n* = 237)**										
No (*n* = 71)	Ref.		Ref.		Ref.		Ref.		Ref.	
yes (*n* = 166)	−0.03	−0.16; 0.10	−0.08	−0.18; 0.01	0.82	−0.08; 1.73	1.33	0.95; 1.86	−0.04	−0.42; 0.32
**Diebetes Mellitus (*n* = 237)**										
No (*n* = 144)	Ref.		Ref.		Ref.		Ref.		Ref.	
Yes (*n* = 93)	0.10	−0.02; 0.23	0.00	−0.08; 0.10	−0.35	−1.20; 0.49	1.30	0.93; 1.82	0.41	0.05; 0.77
**BMI (*n* = 239)**										
Not overweight (*n* = 95)	Ref.		Ref.		Ref.		Ref.		Ref.	
Overweight (*n* = 144)	−0.02	−0.14; 0.10	0.01	−0.07; 0.11	0.14	−0.68; 0.98	0.54	0.38; 0.78	0.09	−0.26; 0.44
**Physical Activity (*n* = 233)**										
Absent/Mild/Moderate (*n* = 197)	Ref.		Ref.		Ref.		Ref.		Ref.	
High Level (*n* = 36)	0.02	−0.14; 0.18	0.04	−0.07; 0.16	0.23	−1.05; 1.52	1.21	0.81; 1.82	−0.22	−0.71; 0.27
**Alcoholic beverage (*n* = 237)**										
No (*n* = 202)	Ref.		Ref.		Ref.		Ref.		Ref.	
Yes (*n* = 35)	−0.04	−0.21; 0.13	0.05	−0.07; 0.17	−0.47	−1.75; 0.80	0.58	0.29; 1.16	−0.46	−0.99; 0.07
**Smoking (*n* = 237)**										
No (*n* = 226)	Ref.		Ref.		Ref.		Ref.		Ref.	
Yes (*n* = 11)	0.05	−0.20; 0.32	0.02	−0.16; 0.21	0.33	−1.44; 2.11	1.68	0.77; 3.65	−0.17	−0.97; 0.61

IC95—confidence interval; B *—coeficients β; IRR **—incidence rate ratio; MW—minimum wage; HF—heart failure; BMI—body mass index.

**Table 2:** Logistic and linear regressions and their respective confidence intervals between the total BALANCE dietary index and by food group, and the clinical variables of patients hospitalized with CHF in Aracaju, SE, 2021.

Logistic Regression										
	Adherence		Green Group		Yellow Group		Blue Group		Red Group	
	OR	CI95	OR	CI95	OR	CI95	OR	CI95	OR	CI95
**NYHA**										
1 and 2	Ref.		Ref.		Ref.		Ref.		Ref.	
3 and 4	0.98	0.89; 1.08	1.14	0.93; 1.40	0.99	0.85; 1.15	1.01	0.84; 1.21	0.87	0.73; 1.02
**EF**										
Changed	Ref.		Ref.		Ref.		Ref.		Ref.	
Preserved	1.05	0.98; 1.13	0.91	0.78; 1.05	1.09	0.98; 1.22	1.00	0.89; 1.13	1.11	0.98; 1.26
**Outcome in 30 days**										
Uneventful	Ref.		Ref.		Ref.		Ref.		Ref.	
Intercurring	0.98	0.92; 1.04	0.95	0.83; 1.09	0.95	0.86; –1.05	1.05	0.94; 1.18	0.98	0.88; 1.10
**Linear regression**										
	β *	**CI95**	β *	**CI95**	β *	**CI95**	β *	**CI95**	β *	**CI95**
**LVESD**	−0.01	−0.06; 0.03	0.05	−0.05; 0.17	−0.09	−0.17; −0.01	0.00	−0.08; 0.96	0.03	−0.06; 0.12
**LVEDD**	−0.00	−0.03; 0.02	0.01	−0.06; 0.08	−0.00	−0.05; 0.05	0.01	−0.04; 0.07	−0.03	−0.10; 0.02

NYHA—New York Heart Association; EF—ejection fraction; LVESD—left ventricular end-systolic diameter; LVEDD—left ventricular end-diastolic diameter. * Adjustment variables: age, sex, alcohol, race, smoking, IPAQ, BMI, hypertension and diabetes.

## Data Availability

The data that support this study can be obtained from: www.ufs.br/Department of Physical Education, accessed on 12 December 2021.
